# Impact of clinical pharmacist-led behavioural theory-based discharge service to promote medication adherence in patients with acute coronary syndrome: a randomised controlled trial

**DOI:** 10.1007/s11096-026-02134-y

**Published:** 2026-04-07

**Authors:** Muhammed Yasir Demirci, Bulent Mutlu, Mesut Sancar, Betul Okuyan

**Affiliations:** 1 Department of Clinical Pharmacy, Faculty of Pharmacy, Marmara University, Maltepe, Istanbul, Türkiye; 2 Department of Cardiology, School of Medicine, Marmara University, Istanbul, Türkiye

**Keywords:** Acute coronary syndrome, Behaviour change techniques, Discharge, Health belief model, Medication reconciliation, Medication review, Medication adherence, Patient counselling

## Abstract

**Introduction:**

Medication non-adherence is common in patients with acute coronary syndrome (ACS) and may increase the risk of cardiac readmissions and mortality.

**Aim:**

To evaluate the effect of a clinical pharmacist-led, behavioural theory-based discharge service designed to promote medication adherence on 30-day cardiac readmissions (primary outcome) and on clinical, humanistic, and healthcare utilisation outcomes over 360 days in patients with ACS.

**Method:**

In this single-centre, parallel-group randomised controlled trial, adult patients hospitalised with ACS were assigned by permuted block randomisation (block size 8) to the intervention or control group. The intervention comprised a clinical pharmacist-led, behavioural theory-based discharge service to promote medication adherence. The intervention consisted of medication reconciliation, medication review and patient counselling based on components of the behaviour change technique taxonomy and Health Belief Model. The primary outcome was 30-day hospital readmission for cardiac reasons. Secondary outcomes included all-cause and cardiac readmissions, emergency department visits, all-cause and cardiac mortality, medication adherence, LDL (low-density lipoprotein) target attainment, and quality of life over 360 days.

**Results:**

A total of 167 patients were analysed (intervention: n = 80; control: n = 87). The primary outcome occurred in 0/80 (0.0%) in the intervention group versus 5/87 (5.7%) in the control group (risk difference − 5.7%, 95% CI − 12.8 to 2.1%; *p* >0.050). Over 360 days, the control group had higher adjusted odds of cardiac readmission (aOR 4.4; 95% CI 1.2–16.0; *p* = 0.027), all-cause readmission (aOR 3.7; 95% CI 1.1–11.7; *p* = 0.029), and non-adherence at 30 days (aOR 2.4; 95% CI 1.1–5.2; *p* = 0.028). At 180 days, the control group had lower adjusted odds of LDL target attainment (aOR 0.4; 95% CI 0.2–0.9; *p* = 0.038).

**Conclusion:**

This intervention reduced 30-day cardiac readmission, but the effect was not statistically significant. According to findings of secondary outcomes, this behavioural theory-based discharge service at discharge might be effective in reducing healthcare utilisation in the long term and improving the short-term target for medication adherence in patients with acute coronary syndrome.

**Trial registration:**

ClinicalTrials.gov NCT05153707.

**Supplementary Information:**

The online version contains supplementary material available at 10.1007/s11096-026-02134-y.

## Impact statements

- A clinical pharmacist-led, behavioural theory-based discharge service designed to promote medication adherence can consist of medication reconciliation, medication review and patient counselling based on components of the behaviour change technique taxonomy and Health Belief Model.
- While the 30-day cardiac readmission primary outcome did not reach statistical significance, a clinical pharmacist-led, behavioural theory-based discharge service to promote medication adherence was associated with lower longer-term health-care utilisation and improved short-term (30-day) self-reported medication adherence.
- Future studies should test whether adding post-discharge follow-up sessions and using designs that can isolate the contribution of theory-based components improve the durability of adherence and other outcomes.

## Introduction

Acute coronary syndrome (ACS) is an acute exacerbation of coronary artery disease and is associated with a high risk of recurrent cardiovascular events and death, particularly during the first year after the index event [1–5]. The 30-day readmission rate is 11–14% among patients with ACS [6]. Secondary prevention, including adherence to cardio-protective medications and healthy lifestyle behaviours, is essential to reduce this risk [7]. However, adherence after discharge is often suboptimal and has been associated with worse outcomes, whereas better adherence is linked to lower readmission and adverse event rates [8–12].

Theory-based approaches are increasingly used to design medication adherence interventions, including pharmacist-led services [13]. The COM-B (Capability, Opportunity, Motivation, and Behaviour) model and behaviour change technique (BCT) taxonomy help identify determinants of non-adherence and select intervention components to address them [14–16]. Patient education based on the Health Belief Model may improve preventive behaviours, and the teach-back method may improve understanding during discharge counselling [17, 18]. Although transition-of-care interventions have been studied in ACS, much of the evidence relates to multidisciplinary or non-pharmacist-led approaches, and the specific contribution of the clinical pharmacist (CP) at discharge remains less clearly defined [19, 20]. Previous ACS discharge interventions have improved medication adherence, but effects on readmissions, mortality, and quality of life have been inconsistent [19–22].

Moreover, pharmacist-involved discharge interventions vary in content and intensity and are often not explicitly theory-based; early cardiac readmissions are inconsistently used as a primary endpoint and longer-term follow-up is variably reported [21, 22]. We therefore designed a structured, CP-led behavioural theory–based discharge service delivered using a written standard operation procedure (including checklist) and teach-back method, with 30-day cardiac readmissions as the primary endpoint and 360-day follow-up to assess longer-term healthcare utilisation and sustained effects.

## Aim

This study evaluated the impact of CP-led behavioural theory-based discharge service to promote medication adherence primarily on 30-day cardiac readmissions and secondary on clinical, humanistic, and healthcare utilisation outcomes over 360 days in patients with ACS.

## Methods

### Study design, participants and setting

This prospective parallel randomised controlled study recruited eligible patients from the cardiology ward of a tertiary university hospital between 15 January 2021 and 15 June 2021. The study protocol was approved by Marmara University School of Medicine Clinical Trials Ethical Committee (Date: December 25th, 2020; protocol number: 2020-09.2020.1321). The study protocol was submitted to ClinicalTrials.gov (NCT05153707) on November 25, 2021, after recruitment of patients, because of technical issues. No amendments were made to the protocol for eligibility criteria and recruitment of patients. This study was reported based on the CONSORT checklist [23] and TIDieR checklist [24] (supplementary files).

Adult patients (18 years and older) admitted to a coronary care unit with ACS (including ST-elevation myocardial infarction (STEMI) and non-ST-elevation myocardial infarction (NSTEMI) were included. All participants provided written informed consent. Patients who did not have consent, were discharged on nights and weekends, transferred to another hospital or ward, refused the treatment, died during hospitalisation, older than 80 years, and had active malignancy were excluded.

### Randomisation

Patients were screened for eligibility by the CP. Eligible patients were allocated 1:1 using a computer-based permuted block randomisation list (block size 8). The randomisation sequence was generated prior to recruitment and stored electronically. The CP generated the allocation sequence, enrolled participants, and implemented allocation by retrieving the next sequential assignment from the electronic list.

### Blinding

Due to the nature of the intervention, participants and the CP delivering the discharge service could not be blinded. Outcome assessment was not blinded to allocation. To minimise contamination, the education session was delivered individually in a separate counselling room rather than at the bedside, and written counselling materials were not shared with control patients.

### Sample size

The study sample size was calculated based on the primary outcome (30-day hospital readmission for cardiac reasons) using G*Power (version 3.1.1). Based on Budiman et al. [25], an absolute between-group difference of 14 percentage points in the rate of 30-day cardiac readmission was assumed. Allowing for 20% attrition, a total sample size of 168 participants (84 per group) was required to achieve 80% power at a two-sided alpha of 0.05.

### Intervention

CP provided medication reconciliation and medication review at discharge, and behavioural theory-based discharge education and counselling session in person on weekdays.

Behavioural theory-based discharge education and counselling session were delivered by a CP who had completed 2 years of postgraduate clinical pharmacy specialist training, including multiple clinical rotations (cardiology among others), and had received online training in the teach-back method for patient education.

**Fig. 1:**
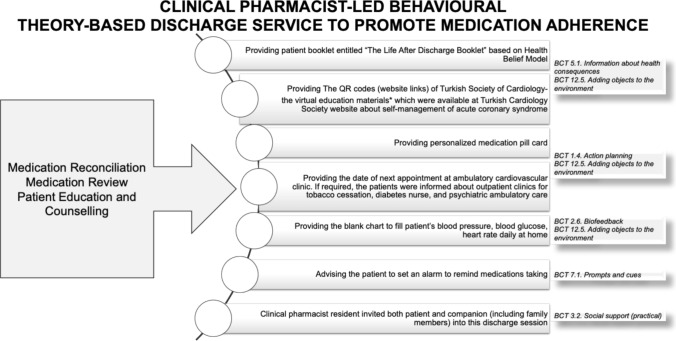
CP-led behavioural theory-based discharge service components (according to the behaviour change technique [BCT] taxonomy v1) *[Available from: https//www.kalbinidinlesen.com/kalp-hastaliklari-ile-yasamak/koroner-kalp-hastaligi-ve-kalp-krizi*in Turkish*] Accessed date 12 July 2023]

This service included the BCT taxonomy [15] selected from previous systematic reviews on the BCTs for patients with ACS [16, 26, 27] and pharmacist-led services [28–30]. The COM-B model was used to select BCTs to promote medication adherence [28–30]. The selected behaviour change techniques were assessed by the research team based on the APEASE (Affordability, Practicability, Effectiveness, Acceptability, Side effects/safety, and Equity) criteria [31]. The COM-B model was used to identify patient-specific adherence barriers across capability (knowledge/skills to manage medicines), opportunity (practical supports and cues), and motivation, and to guide selection of BCT components to target these barriers. The Health Belief Model informed the educational content to strengthen reflective motivation by addressing perceived susceptibility and severity, and consequences of non-adherence, and reinforcing the benefits of regular medicine use and lifestyle change. CP-led Behavioural Theory Based Discharge Service (Fig. 1) was reported using the TIDieR checklist [24] (supplementary files).

According to the obtained best possible medication history taken during the medication reconciliation at discharge, a personalised medication pill card was provided to each patient (including medication name, indication, dose, frequency and clinically significant adverse drug reactions) after medication review. The CP scheduled medication administration with the patient by providing a personalised medication pill card.

The patient booklet entitled “The Life After Discharge Booklet” was developed by using the Health Belief Model to promote medication adherence (susceptibility [rate of non-adherence to medication in patients with ACS] and severity (how non-adherence to medications could impact prognosis of their health conditions), benefit (benefits of taking their medications regularly), and threat (outcomes of non-adherence to their medications), and lifestyle changes (diet, smoking, physical activity) [17]. The booklet was given to the patients during this session. Turkish Society of Cardiology- the virtual education materials were sent to patients and/or companions by text message on day of discharge. Each patient was provided with a blank chart to record daily blood pressure, blood glucose, and heart rate at home.

During the intervention, patients’ companions participated in this session to promote patients’ motivation on healthy behaviours (including medication adherence and lifestyle changes). The next appointment date at the ambulatory cardiovascular clinic was provided. If required, patients were informed about outpatient clinics for tobacco cessation, diabetes nurse, and psychiatric ambulatory care.

The behavioural theory-based discharge education and counselling session was conducted by using the teach-back method.

An intervention standard operation procedure (including checklist) for CP was prepared. All the study materials were reviewed by an expert panel (cardiology physicians, nurses and CPs). For each intervention, the CP completed the checklist. All patients’ materials were tested for face validity during a pilot study among patients with ACS. The single session was completed in approximately 20 min.

The control group received routine care by physicians and nurses during hospitalisation and at discharge. In routine care, there were no structured medication reconciliation, medication review, and education and counselling session services at the hospital, and no pharmacist was involved in this usual discharge care.

### Outcomes

The primary objective was to evaluate the impact of the CP-led behavioural theory-based discharge service on 30-day cardiac re-admission in patients with ACS. Readmission outcomes were ascertained from the hospital's electronic records. Cardiac readmission was classified by the documented primary reason and/or diagnosis for readmission.

Secondary outcomes were all-cause and cardiac re-admission, emergency department visits, all-cause and cardiac mortality, medication adherence, LDL target attainment, and quality of life over 360 days. Medication adherence to cardio-protective medications (aspirin, ticagrelor, clopidogrel, statins, beta-blockers, angiotensin-converting enzyme [ACE] inhibitors/angiotensin receptor blocker [ARB], proton pump inhibitors [PPI]) was assessed over 1 year using the Turkish version of the Medication Adherence Report Scale (MARS) [32–34]. MARS assessed adherence through 5-item, total score ranging from 1 to 5. If the total score was 5, this categorised as adherent to medications [35]. LDL target attainment was assessed at 6 months, and 12 months. Change in quality of life was assessed with the EuroQol questionnaire (EQ-5D-3L), including Quality-based index and VAS scores, over 1 year [36–38]

Patient characteristics (including age, sex, education year, number of chronic diseases, Charlson Comorbidity Index, body mass index, smoking, reason for admission, history of cardiovascular heart disease, type 2 diabetes mellitus, and hypertension) were collected. The health literacy was assessed by using the Single Item Literacy Screen [39, 40]. Harms or unintended effects related to the intervention were not systematically collected. However, any intervention-related adverse events spontaneously reported by participants were planned to be recorded.

### Statistical analysis

All data analyses were performed using SPSS version 11.0. Continuous variables were summarised as mean (standard deviation [SD]) or median (25th–75th percentiles), and categorical variables as number (%). Normality was assessed using the Kolmogorov–Smirnov and Shapiro–Wilk tests, where appropriate. Between-group comparisons were performed using the independent-samples t-test or Mann–Whitney U test for continuous variables (as appropriate) and the chi-square test (or Fisher’s exact test when expected cell counts were small) for categorical variables. For dichotomous outcomes, unadjusted odds ratios (ORs) with 95% confidence intervals (CIs) were calculated. Adjusted ORs were estimated using the Mantel–Haenszel method stratified by sex (female/male) to account for the baseline imbalance in sex. Analyses were performed according to the intention-to-treat principle. Patient-reported outcomes were analysed using available-case data at each follow-up time point. Numbers needed to treat (NNTs) were calculated from absolute risk differences for selected outcomes. Analyses were two-sided, and *p* < 0.05 was considered statistically significant. Sensitivity analyses were performed to assess the potential impact of missing data; results were consistent with the primary analyses.

### Ethics approval

The study protocol was approved by Marmara University School of Medicine Clinical Trials Ethical Committee (Date: December 25th, 2020; Protocol no: 2020-09.2020.1321). All participants signed an informed consent form.

## Results

**Fig. 2:**
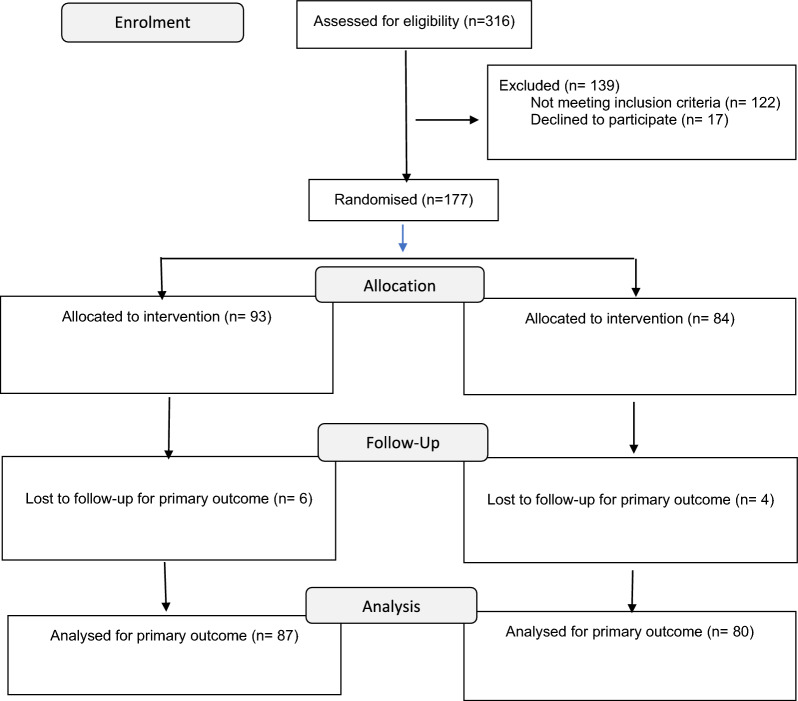
Study flow diagram

In this study, 316 patients were considered for recruitment, and 167 patients were analysed (intervention n = 80; control n = 87) (Fig. 2). The participant flow chart is presented in Fig. 2. Baseline patient characteristics by group are presented in Table 1.

**Table 1:** Characteristics of patients among groups at baseline

	Control group (n = 87)	Study group (n = 80)
Age mean (SD)	56.6 (9.5)	55.9 (10.1)
Sex n (%)		
Female	11 (12.6)	21 (26.3)
Man	76 (87.4)	59 (73.7)
Education level* n (%)		
8 years or higher	25 (29.1)	23 (29.1)
< 8 years	62 (70.9)	57 (70.9)
Low health literacy n (%)		
Yes	39 (53.4)	40 (56.3)
Number of chronic diseases median [25th–75th percentile]	1.0 [0.0–2.0]	1.0 [0.0–2.0]
Charlson comorbidity index median [25th–75th percentile]	3.0 [2.0–5.0]	3.0 [2.0–5.0]
BMI median [25th–75th percentile]	27.9 [26.0–31.7]	28.8 [25.7–31.1]
Smoking n (%)		
Yes	46 (52.9)	48 (60.0)
Reason of admission n (%)		
STEMI	54 (62.1)	48 (60.0)
NSTEMI	33 (37.9)	32 (40.0)
History of CHD n (%)		
Yes	25 (28.7)	16 (20.0)
Type 2 DM n (%)		
Yes	23 (26.4)	22 (27.5)
Hypertension n (%)		
Yes	28 (32.2)	29 (36.3)

SD: Standard deviation; BMI: Body mass index; CHD: Coronary heart disease; DM: Diabetes mellitus; STEMI: ST-elevation myocardial infarction; NSTEMI: Non-ST-elevation myocardial infarction

*Group classified according to obligatory education year. Baseline characteristics are presented descriptively by group; no statistical tests were performed for baseline comparisons, in line with CONSORT guidance

The rate of readmission (including all-cause and cardiac), emergency department visits, and deaths among groups during the study period is presented in Table 2. The primary outcome (30-day cardiac readmission) occurred in 0/80 (0.0%) patients in the study group and 5/87 (5.7%) patients in the control group; this difference did not reach statistical significance (*p* >0.050), indicating statistical uncertainty due to the small number of events. Compared with the control group (14.9%), the rate of 360-day readmission for cardiac reasons was statistically lower in the study group (3.8%) (*p* < 0.05). Compared with the control group (16.1%), the rate of 360-day readmission for all causes was statistically lower in the study group (5.0%) (*p* < 0.05). In the study group, there were no all-cause and cardiac deaths within 360 days after discharge. In the control group, four patients died due to cardiac reasons and six patients died due to all causes within 360 days after discharge.

**Table 2:** Rate of readmission (including all-cause and cardiac), emergency department visits, and deaths during the study period

	Control group (n = 87)	Study group (n = 80)	*p*-value
Primary outcome			
30-day cardiac readmission n (%)			
Yes	5 (5.7)	0 (0.0)	>0.05
Secondary outcome			
Cardiac readmission			
90-day cardiac readmission n (%)			
Yes	7 (8.1)	3 (3.8)	0.332
180-day cardiac readmission n (%)			
Yes	9 (10.5)	3 (3.8)	0.171
360-day cardiac readmission n (%)			
Yes	13 (14.9)	3 (3.8)	0.023
All-cause readmission			
30-day all-cause readmission n (%)			
Yes	5 (5.7)	1 (1.3)	0.253
90-day all-cause readmission n (%)			
Yes	7 (8.1)	4 (5.0)	0.617
180-day all-cause readmission n (%)			
Yes	9 (10.5)	4 (5.0)	0.307
360-day all-cause readmission n (%)			
Yes	14 (16.1)	4 (5.0)	0.032
30-day emergency department visit n (%)			
Yes	14 (16.1)	12 (15.0)	1
90-day emergency department visit n (%)			
Yes	17 (19.8)	16 (20.0)	1
180-day emergency department visit n (%)			
Yes	19 (22.1)	18 (22.5)	1
360-day emergency department visit n (%)			
Yes	30 (36.6)	24 (30.0)	0.47
360-day all-cause mortality n (%)			
Yes	6 (6.9)	0 (0.0)	–
360-day cardiac mortality n (%)			
Yes	4 (4.6)	0 (0.0)	–

*p-values for mortality outcomes are not presented because event counts were small (including zero cells); mortality results are shown descriptively

Assessments of the rate of medication adherence and LDL target attainment, and quality of life are presented in Table 3. At 30 days, the medication adherence was higher in the intervention group than in the control group (85.0% vs 69.9%; *p* = 0.034). LDL target attainment was higher in the intervention group at 180 days (82.4% vs 63.5%) and the between-group comparison was borderline and did not reach statistical significance (*p* = 0.053). Quality-of-life outcomes (EQ-5D-3L index and VAS) were similar between groups at all follow-up assessments, with no statistically significant differences observed in QoL during follow-up (Table 3 and Supplementary) (all *p* > 0.05).

**Table 3:** Assessments of the rate of adherence to medication and achieving LDL target attainment, and quality of life during the study period

	Control group (n = 87)	Study group (n = 80)	*p*-value
Medication adherence			
30-day adherence			
n (%)			
Adherent patient	58 (69.9)	68 (85.0)	0.034
Missing data	4	0	
90-day adherence			
Adherent patient	34 (69.4)	35 (72.9)	0.873
Missing data	38	32	
180-day adherence			
Adherent patient	30 (53.6)	37 (69.8)	0.122
Missing data	31	27	
360-day adherence			
Adherent patient	37 (49.3)	40 (57.1)	0.346
Missing data	12	10	
LDL target attainment			
180-day LDL target attainment n (%)			
Yes	33 (63.5)	42 (82.4)	0.053
Missing data	35	49	
360-day LDL target attainment n (%)			
Yes	18 (50.0)	26 (66.3)	0.219
Missing data	51	41	
EQ-5D-3L index			
30-day EQ-5D-3L index			
Median [IQR]	0.1 [0.0–0.2]	0.1 [0.0–0.3]	0.987
90-day EQ-5D-3L index			
Median [IQR]	0.1 [0.0–0.2]	0.1 [0.0–0.2]	0.692
180-day EQ-5D-3L index			
Median [IQR]	0.0 [0.0–0.1]	0.0 [0.0–0.1]	0.928
360-day EQ-5D-3L index			
Median [IQR]	0.0 [0.0–0.1]	0.0 [0.0–0.1]	0.871
EQ-5D-3L VAS			
30-day VAS			
Median [IQR]	70.0 [60.0–85.0]	70.0 [60.0–90.0]	0.733
90-day VAS			
Median [IQR]	75.0 [60.0–90.0]	79.0 [62.5–90.0]	0.928
180-day VAS			
Median [IQR]	80.0 [60.0–90.0]	80.0 [70.0–90.0]	0.52
360-day VAS			
Median [IQR]	77.5 [65.0–86.2]	75.0 [60.0–87.5]	0.19

IQR: Interquartile rate; VAS:, Visual analogue scale

Odds ratios and adjusted odds ratios for outcomes are presented in Table 4. NNT for 360-day all-cause readmission between the study group and control group was found to be 8.3. NNT for 360-day cardiac readmission between the study group and control group was found to be 8.3. NNT for 30-day adherence between the study group and control group was found to be 6.6. NNT for 180-day LDL target attainment between the control group and study group was found to be 5.3. Over 360 days, the control group had higher adjusted odds of cardiac readmission (aOR 4.4; 95% CI 1.2–16.0; *p* = 0.027), all-cause readmission (aOR 3.7; 95% CI 1.1–11.7; *p* = 0.029), and non-adherence at 30 days (aOR 2.4; 95% CI 1.1–5.2; *p* = 0.028). At 180 days, the control group had lower adjusted odds of LDL target attainment (aOR 0.4; 95% CI 0.2–0.9; *p* = 0.038). No intervention-related adverse events were identified from participant reports or routine clinical documentation during follow-up.

**Table 4:** Findings of the Odds ratio and the adjusted Odds ratio of outcomes

	OR	95% CI	*p* value	Adjusted OR*	95% CI	*p-*value
360-day all-cause readmission						
Control group	3.8	1.2–12.1	0.024	3.7	1.1–11.7	0.029
Study group	1			1		
360-day cardiac readmission						
Control group	4.7	1.3–17.2	0.019	4.4	1.2–16	0.027
Study group	1			1		
30-day medication non-adherence						
Control group	2.4	1.1–5.3	0.023	2.4	1.1–5.2	0.028
Study group	1			1		
180-day LDL target attainment						
Control group	0.4	0.2–0.9	0.034	0.4	0.2–0.9	0.038
Study group	1			1		

*Adjusted for sex using the Mantel–Haenszel method

## Discussion

This randomised controlled trial evaluated the impact of CP-led discharge service on patients with ACS. In this study, more patients in the control group were readmitted to the hospital within 30 days for cardiac reasons than in the intervention group. Although the primary outcome did not reach statistical significance. Given the small number of events, the trial may have been underpowered to detect a difference in early readmissions, and larger trials are needed to confirm clinical benefit. Adjusted odds ratios of 360-day readmission for cardiac reasons and all-cause, and 30-day non-adherence were statistically lower, and the adjusted odds ratio of 180-day LDL target attainment was statistically higher in the study group. This pattern might be consistent with the intervention’s more pronounced early impact on medication adherence, which could translate into improved lipid goal attainment at 6 months.

Evidence on pharmacist-involved discharge and transition-of-care interventions after ACS is mixed, and interpretation depends on study design, comparability of interventions, and outcomes assessed. Observational evidence has suggested potential reductions in readmissions after implementation of pharmacist-led discharge programmes; for example, a retrospective pre–post cohort reported a reduction in 90-day all-cause readmission [41]. However, such non-randomised designs are more susceptible to confounding. Therefore, effect estimates should be interpreted cautiously against randomised controlled trial evidence. In contrast, randomised studies of post-discharge pharmacy services have reported mixed findings for early readmission endpoints, with one trial showing no statistically significant difference in readmissions despite numerical trends [42].

Prior pharmacist-involved ACS interventions, as well as other discharge education strategies, have reported improvements in adherence in early months after discharge, but effects on QoL, mortality and readmissions have often been non-significant over short follow-up [20, 21]. Studies also differ in the comparator. For example, a “routine care” arm in prior studies may still include pharmacist education/counselling, which reduces contrast and limits direct comparability with our setting, where usual care did not include a structured pharmacist-led service [43]. Moreover, discrepancies between self-reported adherence and objective measures have been highlighted in pharmacist service studies [44], which is relevant to interpreting our patient-reported adherence findings. Across studies, differences in intervention content, comparison with usual care, and outcome definitions likely contribute to variation in reported effects, reinforcing the need for clearly defined outcomes and adequately powered trials.

Future studies could enhance the impact of this service by adding follow-up sessions led by a CP in an ambulatory care setting. These follow-up sessions could be delivered in-person or by phone at an ambulatory care clinic or in a community pharmacy setting by trained pharmacists [45]. During these follow-up sessions, current barriers to medication adherence could be identified and addressed using a recently developed, structured, theory-based toolkit [46].

## Strengths and limitations

This study has several strengths, including the randomised controlled design, and behavioural theory–based intervention, delivered in a structured manner using a written standard operation procedure (including teach-back) to support intervention fidelity and reproducibility. Additionally, outcomes were assessed across multiple domains (health-care utilisation, clinical and humanistic outcomes) with follow-up extending to 360 days, providing insight into both short- and longer-term patterns after discharge.

This study has several limitations, particularly in relation to the primary outcome. In addition, the observed 30-day cardiac readmission rate in the control group was lower than the rate assumed for the sample size calculation, and the small number of events yields wide uncertainty around the true effect, increasing the risk of type II error. Accordingly, the non-significant primary outcome should be interpreted cautiously and confirmed in future adequately powered trials. The same CP generated the allocation sequence, enrolled participants, implemented allocation, and delivered the intervention, and allocation concealment was not fully maintained because the allocation list could be accessed during recruitment; therefore, selection bias is one of the limitations.

A baseline imbalance in sex distribution occurred between groups. We addressed this using Mantel–Haenszel stratification by sex for dichotomous outcomes, but residual confounding cannot be ruled out, and the study was not powered to assess effect modification. The intervention was delivered as a bundle of clinical pharmacy activities comprising medication reconciliation and review at discharge, and behavioural theory-based discharge counselling, so the contribution of individual components cannot be isolated; findings therefore reflect the overall CP-led discharge service rather than discharge counselling alone. Generalisability may be limited by the single-centre setting and delivery by a CP. Adherence and QoL were self-reported and may be subject to interviewer and social-desirability bias, and longer-term follow-up was affected by increasing missing data after 90 days. Finally, the trial was registered after recruitment had completed; although outcomes and analyses were pre-specified in the protocol prior to data analysis, retrospective registration may raise concerns about selective reporting and were considered when interpreting the findings.

## Conclusion

This study demonstrated that a behavioural theory-based patient counselling service may be effective in reducing healthcare utilisation in the long term and improving short-term medication adherence in patients with ACS. Future studies should be designed to evaluate the incremental contribution of the theory-based components and tested in larger, adequately powered trials. These data support the role of CPs as part of an interprofessional team in ACS management and suggest that discharge education should be considered a fundamental component of the treatment plan for this patient group. Further modification of the service will be considered by adding follow-up sessions at the primary care.

## Supplementary Information

Below is the link to the electronic supplementary material.

Supplementary file1 (DOCX 46 KB)

## Data Availability

The dataset is available from the corresponding author upon reasonable request.
