# Supplementary material for: Impact of clinical pharmacist-led behavioural theory-based discharge service to promote medication adherence in patients with acute coronary syndrome: a randomised controlled trial
Source: Int J Clin Pharm. 2026 Apr 7;48(4):1548–58. doi: 10.1007/s11096-026-02134-y (PMC13369226; doi:10.1007/s11096-026-02134-y)
Supplement: Supplementary file 1 — Supplementary file1 (DOCX 46 KB) [file 11096_2026_2134_MOESM1_ESM.docx]

**Impact of Clinical Pharmacist-led Behavioural Theory-Based Discharge Service to Promote Medication Adherence in Patients with Acute Coronary Syndrome: A Randomised Controlled Trial**

Muhammed Yasir Demirci, Bulent Mutlu, Mesut Sancar, Betul Okuyan

**Authors**

M. Yasir Demirci (BSc), ORCID ID: 0000-0003-3050-5782

Department of Clinical Pharmacy, Faculty of Pharmacy, Marmara University, 34854, Istanbul, Türkiye

Bulent Mutlu (MD), Professor, ORCID ID: 0000-0003-0973-3422

Department of Cardiology, School of Medicine, Marmara University, 34854 Istanbul, Türkiye

Mesut Sancar (PhD), Professor, ORCID ID: 0000-0002-7445-3235

Department of Clinical Pharmacy, Faculty of Pharmacy, Marmara University, 34854 Istanbul, Türkiye

Betul Okuyan (PhD), Associate Professor, ORCID ID: 0000-0002-4023-2565

Department of Clinical Pharmacy, Faculty of Pharmacy, Marmara University, 34854 Istanbul, Türkiye

**Corresponding author:**

* Correspondence: Betul Okuyan (PhD), Associate Professor, ORCID ID: 0000-0002-4023-2565

Clinical Pharmacy Department, Faculty of Pharmacy, Marmara University, Istanbul, Türkiye.

Marmara University, Faculty of Pharmacy, Maltepe Istanbul, Türkiye.

e-mail address: [betulokuyan@yahoo.com](mailto:betulokuyan@yahoo.com)

**Supplement Files**

**S-1** Findings of Dimensions of EQ-5D-3L

|  | **Level** | | | ***Total*** | ***p-value*** |
| --- | --- | --- | --- | --- | --- |
|  | **1** | **2** | **3** |  |  |
| **Mobility** |  |  |  |  |  |
| **30-days mobility**  *Control*  *Study Group* | 60 (72.3)  58 (72.5) | 22 (26.5)  17 (21.3) | 1 (1.2)  5 (6.3) | 83  80 | 0.193 |
| **90-days mobility**  *Control*  *Study Group* | 38 (77.6)  32 (69.6) | 11 (22.4)  11 (23.9) | 0 (0)  3 (6.5) | 47  46 | 0.181 |
| **180-days mobility**  *Control*  *Study Group* | 46 (83.6)  41 (77.4) | 9 (16.4)  12 (22.6) | 0 (0)  0 (0) | 55  53 | 0.561 |
| **360-days mobility**  *Control*  *Study Group* | 67 (90.5)  52 (74.3) | 7 (9.5)  14 (20.0) | 0 (0)  4 (5.7) | 74  70 | 0.017 |

**Self-Care**

| **30-days self-care**  *Control*  *Study Group* | 80 (96.4)  71 (88.8) | 3 (3.6)  5 (6.3) | 0 (0)  4 (5.0) | 83  80 | 0.083 |
| --- | --- | --- | --- | --- | --- |
| **90-days self-care**  *Control*  *Study Group* | 47 (95.9)  40 (88.9) | 2 (4.1)  3 (6.7) | 0 (0)  2 (4.4) | 49  45 | 0.273 |
| **180-days self-care**  *Control*  *Study Group* | 52 (94.5)  51 (92.7) | 3 (5.5)  4 (7.3) | 0 (0)  0 (0) | 55  55 | 1.0 |
| **360-days self-care**  *Control*  *Study Group* | 72 (97.3)  65 (91.5) | 2 (2.7)  3 (4.2) | 0 (0)  3 (4.2) | 74  71 | 0.174 |

**Usual Activities**

| **30-days usual activities**  *Control*  *Study Group* | 63 (79.7)  56 (73.7) | 9 (11.4)  10 (13.2) | 7 (8.9)  10 (13.2) | 79  76 | 0.626 |
| --- | --- | --- | --- | --- | --- |
| **90-days usual activities**  *Control*  *Study Group* | 38 (79.2)  28 (68.3) | 5 (10.4)  8 (19.5) | 5 (10.4)  5 (12.2) | 48  41 | 0.434 |
| **180-day usual activities**  *Control*  *Study Group* | 43 (81.1)  38 (76.0) | 8 (15.1)  11 (22.0) | 2 (3.8)  1 (2.0) | 53  50 | 0.598 |
| **360-days usual activities**  *Control*  *Study Group* | 59 (83.1)  50 (75.8) | 10 (14.1)  9 (13.6) | 2 (2.8)  7 (10.6) | 71  66 | 0.183 |

**Pain/Discomfort**

| **30-days pain / discomfort**  *Control*  *Study Group* | 59 (71.1)  55 (68.8) | 20 (24.1)  14 (17.5) | 4 (4.8)  11 (13.8) | 83  80 | 0.110 |
| --- | --- | --- | --- | --- | --- |
| **90-days pain / discomfort**  *Control*  *Study Group* | 33 (67.3)  35 (76.1) | 13 (26.5)  6 (13.0) | 3 (6.1)  5 (10.9) | 49  46 | 0.218 |
| **180-days pain / discomfort**  *Control*  *Study Group* | 45 (81.8)  40 (74.1) | 6 (10.9)  9 (16.7) | 4 (7.3)  5 (9.3) | 55  55 | 0.608 |
| **360-days pain / discomfort**  *Control*  *Study Group* | 58 (78.4)  58 (84.1) | 9 (12.2)  5 (7.2) | 7 (9.5)  6 (8.7) | 74  69 | 0.593 |

**Anxiety/Depression**

| **30-days anxiety / depression**  *Control*  *Study Group* | 58 (69.9)  53 (67.1) | 19 (22.9)  18 (22.8) | 6 (7.2)  8 (10.1) | 83 | 0.803 |
| --- | --- | --- | --- | --- | --- |
| **90-days anxiety / depression**  *Control*  *Study Group* | 37 (75.5)  29 (63.0) | 10 (20.4)  14 (30.4) | 2 (4.1)  3 (6.5) | 49  46 | 0.418 |
| **180-days anxiety / depression**  *Control*  *Study Group* | 44 (80.0)  45 (81.8) | 8 (14.5)  6 (10.9) | 3 (5.5)  4 (7.3) | 55  55 | 0.803 |
| **360-days anxiety / depression**  *Control*  *Study Group* | 65 (87.8)  59 (83.1) | 8 (10.8)  7 (9.9) | 1 (1.4)  5 (7.0) | 74  71 | 0.227 |

**S-2** The TIDieR (Template for Intervention Description and Replication) Checklist

**
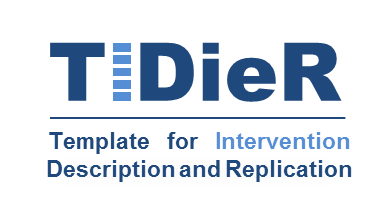
The TIDieR (Template for Intervention Description and Replication) Checklist*:**

Information to include when describing an intervention and the location of the information

| **Item number** |  |  |
| --- | --- | --- |
|  | **Item** | **Where located **** |
|  |  |  |
|  | **BRIEF NAME** |  |
| **1.** | Provide the name or a phrase that describes the intervention. | Clinical Pharmacist-led Service for Acute Coronary Syndrome (ACS) |
|  | **WHY** |  |
| **2.** | Describe any rationale, theory, or goal of the elements essential to the intervention. | Clinical and patient-reported outcomes (including medication adherence, quality of life) are poor in patients with acute coronary syndrome (ACS) after hospital discharge. Although there are education and counselling intervention studies among patients with ACS, the impact of these interventions on clinical and patient-reported outcomes is unclear. Theory based intervention was found to be more effective than pragmatic interventions. Behavioral theory-based discharge services might increase medication adherence and patient-reported outcomes among patients with acute coronary syndrome. |
|  | **WHAT** |  |
| **3.**  **4.** | Materials: Describe any physical or informational materials used in the intervention, including those provided to participants or used in intervention delivery or in training of intervention providers. Provide information on where the materials can be accessed (e.g. online appendix, URL).  Procedures: Describe each of the procedures, activities, and/or processes used in the intervention, including any enabling or support activities. | Clinical pharmacist has provided medication reconciliation, and medication review at discharge, and behavioural theory-based discharge education and counselling session in person.  Personalized medication pill card: The medication reconciliation and medication review services were delivered. According to obtained best possible medication history during the medication reconciliation service at discharge, and a personalized medication pill card was provided to each patient (including medication name, indication, dose and frequency, and clinically significant adverse drug reactions) after conducting medication review service. Clinical pharmacist scheduled when and how to administer medications with the patient by providing a personalized medication pill card (1.4. Action planning and 12.5. Adding objects to the environment) and advised the patient to set an alarm (7.1. Prompts and cues).  The patient booklet: The behavioural theory-based discharge education and counselling session was conducted by using teach back method. The patient booklet entitled The Life After Discharge Booklet and visual education materials (including Turkish educational videos supported by Turkish National Cardiology Society) were used as a guidance during this session (12.5. Adding objects to the environment). This booklet was developed by using the Health Belief Model to promote medication adherence and lifestyle changes (diet, smoking, physical activity) (5.1. Information about health consequences).  The QR codes (website links) of Turkish Society of Cardiology- the virtual education materials were provided [Available from: https//www.kalbinidinlesen.com/kalp-hastaliklari-ile-yasamak/koroner-kalp-hastaligi-ve-kalp-krizi] Accessed date 12 July 2023], which were available at the Turkish Cardiology Society website about self-management of acute coronary syndrome (12.5. Adding objects to the environment).  The blank chart to fill in patient’s blood pressure, blood glucose, and heart rate daily at home was provided for each patient (12.5. Adding objects to the environment and 2.6 Biofeedback). The date of the next appointment at the ambulatory cardiovascular clinic was provided as well (1.4. Action planning). If required, the patients were informed about outpatient clinics for tobacco cessation, diabetes nurse, and psychiatric ambulatory care (1.4. Action planning).  Clinical pharmacist resident invited both patient and companion (including family members) into the session (3.2. Social support (practical).  Patient education and counselling are provided in a private room. The patient booklet was given the patients in person during patient education and counselling session. The QR codes (website links) of Turkish Society of Cardiology- the virtual education materials were sent to patients and/or family caregivers by a phone message on the discharge day of patients. The personalized medication pill card was prepared according to the best possible medication history taken during medication reconciliation at discharge. The blank chart to fill patient’s blood pressure, blood glucose, and heart rate daily at home was provided for each patient. |
|  | **WHO PROVIDED** |  |
| **5.** | For each category of intervention provider (e.g. psychologist, nursing assistant), describe their expertise, background and any specific training given. | Patient education and counselling were delivered by a clinical pharmacist resident who had completed two years of postgraduate clinical pharmacy specialist training, including multiple clinical rotations (cardiology among others), and had received online training in the teach-back method for patient education. |
|  | **HOW** |  |
| **6.** | Describe the modes of delivery (e.g. face-to-face or by some other mechanism, such as internet or telephone) of the intervention and whether it was provided individually or in a group. | This service was performed in person with the patient and companion. During the intervention patients’ companions participated in this patient education and counselling session to promote patients’ motivation for healthy behaviours (including adherence to medication and follow lifestyle changes). |
|  | **WHERE** |  |
| **7.** | Describe the type(s) of location(s) where the intervention occurred, including any necessary infrastructure or relevant features. | This service was provided in the tertiary university hospital of the cardiology department. There was a private room to provide education and counselling sessions. |
|  | **WHEN and HOW MUCH** |  |
| **8.** | Describe the number of times the intervention was delivered and over what period of time including the number of sessions, their schedule, and their duration, intensity or dose. | The single session was completed approximately 20 minutes. |
|  | **TAILORING** |  |
| **9.** | If the intervention was planned to be personalised, titrated or adapted, then describe what, why, when, and how. | This service was personalized according to the patient's medication pill card. Mainly, the patient education and counselling for management of post-acute coronary syndrome were generally the same for all patients. Patients’ other medications for comorbidities (such as type 2 diabetes) were also included on their discharge pill card, and information was also provided for these medications. |
|  | **MODIFICATIONS** |  |
| **10.^ǂ^** | If the intervention was modified during the course of the study, describe the changes (what, why, when, and how). | There were no amendments and modifications in this service. |
|  | **HOW WELL** |  |
| **11.** | Planned: If intervention adherence or fidelity was assessed, describe how and by whom, and if any strategies were used to maintain or improve fidelity, describe them. | An intervention standard operating procedure (including checklists) for the clinical pharmacist was prepared for this study. This checklist was reviewed by an expert panel (cardiology physicians, nurses and pharmacists). In every intervention, the clinical pharmacist resident completed the checklist. |
| **12.^ǂ^** | Actual: If intervention adherence or fidelity was assessed, describe the extent to which the intervention was delivered as planned. | All participants were adherent to receive this session. There was no dropout during this session. |
